# Partial Substitution of Meat with Insect (*Alphitobius diaperinus*) in a Carnivore Diet Changes the Gut Microbiome and Metabolome of Healthy Rats

**DOI:** 10.3390/foods10081814

**Published:** 2021-08-05

**Authors:** Sofie Kaas Lanng, Yichang Zhang, Kristine Rothaus Christensen, Axel Kornerup Hansen, Dennis Sandris Nielsen, Witold Kot, Hanne Christine Bertram

**Affiliations:** 1 Department of Food Science, Aarhus University, Agro Food Park 48, 8200 Aarhus, Denmark; sofiekaaslanng@food.au.dk; 2 CiFOOD, Centre for Innovative Food Research, Aarhus University, 8200 Aarhus, Denmark; 3 Department of Food Science, University of Copenhagen, Rolighedsvej 30, 1958 Frederiksberg, Denmark; yichang@food.ku.dk (Y.Z.); dn@food.ku.dk (D.S.N.); 4 Department of Veterinary and Animal Sciences, University of Copenhagen, Grønnegårdsvej 15, 1958 Frederiksberg, Denmark; krs@sund.ku.dk (K.R.C.); akh@sund.ku.dk (A.K.H.); 5 Department of Plant and Environmental Sciences, University of Copenhagen, Thorvaldsensvej 40, 1871 Frederiksberg, Denmark; wk@plen.ku.dk

**Keywords:** NMR-based metabolomics, microbiota, insect protein, protein digestion, alternative proteins

## Abstract

Insects are suggested as a sustainable protein source of high nutritional quality, but the effects of insect ingestion on processes in the gastrointestinal tract and gut microbiota (GM) remain to be established. We examined the effects of partial substitution of meat with insect protein (*Alphitobius diaperinus*) in a four-week dietary intervention in a healthy rat model (*n* = 30). GM composition was characterized using’ 16S rRNA gene amplicon profiling while the metabolomes of stomach, small intestine, and colon content, feces and blood were investigated by ^1^H-NMR spectroscopy. Metabolomics analyses revealed a larger escape of protein residues into the colon and a different microbial metabolization pattern of aromatic amino acids when partly substituting pork with insect. Both for rats fed a pork diet and rats fed a diet with partial replacement of pork with insect, the GM was dominated by *Lactobacillus*, *Clostridium cluster XI* and *Akkermansia*. However, Bray-Curtis dissimilarity metrics were different when insects were included in the diet. Introduction of insects in a common Western omnivore diet alters the gut microbiome diversity with consequences for endogenous metabolism. This finding highlights the importance of assessing gastrointestinal tract effects when evaluating new protein sources as meat replacements.

## 1. Introduction

The population of the Earth is estimated to rise to 9.6 billion people by 2050 [1] with a resultant increased demand for food. The growing competition for land and water resources affects our ability to produce food and increases our need to minimize the impact of food production on our resources and environment [2]. Especially the production of protein sources leaves a heavy impact on the environment, where meat production has a high impact regarding water consumption and emission of greenhouse gases [3]. Therefore, there is a massive interest in exploring how to limit the effects on the environment when producing protein and concomitantly ensure protein with a high nutritional value. Compared to meat, plant proteins have a lower environmental impact but also a lower protein nutritional quality as some plant-derived proteins do not contain all essential amino acids and might also contain anti-nutritional compounds [4]. Insects have been reco, gnized as a new and alternative animal-based protein source [5]. The production of insect protein has a high feed conversion ratio [6], emits less greenhouse gases [7], uses less water, and requires less land compared to conventional livestock [6,8].

Insects are comparable in protein content to the conventional animal- and plant-based protein-dense foods, e.g., beef, eggs, milk and soy [9]. Moreover, the content of all essential amino acids in insects meets the requirements of WHO [10]. The total protein content as well as the amino acid composition can moreover be modulated by the feeding substrate [11] and also processing [12]. However, in vivo studies investigating the nutritional value of insects and insect protein are sparse. Few studies have investigated the use of insect protein on muscle protein synthesis [13,14] and muscle mass [15], showing insect protein suboptimal compared to whey both in amino acid availability and stimulation of muscle protein synthesis.

A study in pigs revealed lower ileal digestibility of most amino acids for diets including insect meal compared with a control diet and small changes in the metabolome when 10% of the conventional protein was substituted with insect protein [16]. In humans, a randomized controlled trial investigated the postprandial absorption of insect protein in young men and demonstrated that insect protein provides all essential amino acids, but insect protein was found to have lower bioaccessibility of amino acids than whey but similar to soy protein [17]. A two-week human intervention study investigated the effect of daily ingestion of 25 g cricket powder on the fecal microbiome and the metabolome. The insect supplementation was associated with a decrease in the fecal content of short-chain fatty acids, and minor changes in microbiota composition were observed at the species-level [18]. To our knowledge, these two studies are currently the only reported intervention studies focusing on the nutritional value of insects in a Western-type diet.

In the current study, insect protein isolate isolated from *Alphitobius diaperinus* (lesser mealworm) were used as this protein isolate has at high amount of protein (~82%). Moreover, *Alphitobius diaperinus* has been shown to have a high digestibility in rats (91–94%) [19].

In the present study, we investigated the use of insect protein (*Alphitobius diaperinus*) for a partial replacement (~13%) of meat in a conventional meat product. For this purpose, a four-week intervention study in healthy rats was conducted, where effects of partial insect substitution on growth, food consumption, the gut microbiome, as well as the metabolome were examined.

## 2. Materials and Methods

### 2.1. Animals and Diet

Thirty four-week old Sprague–Dawley (NTac:SD) rats (Taconic, Ll. Skensved, Denmark) were individually earmarked and weighed, before being randomly allocated with three animals into each of ten U1400 cages (Tecniplast, Buguggiate, Italy) with Tapvei^®^ aspen bedding and enrichments such as chewing blocks, tunnels, and nesting material (Brogaarden, Lynge, Denmark) at temperatures of 22 ± 2 °C, humidity of 55 ± 10%, air changing 15–20 times/hour and a 12-h light cycle. Health monitoring at the breeder and the experimental facility revealed no reportable infections in the rats [20]. For an adaption period of four days, all rats were fed ad libitum standard chow diet (Altromin 1324, Brogaarden, Denmark) and had free access to water. After the adaption period, the rats were weighted and fecal samples from each rat were obtained. Subsequently, the rats were cage-wise randomly allocated to one of three diets for a four-week intervention period: (1) standard chow diet (Chow) (*n* = 6), (2) insect-substituted pork sausage diet (Insect) (*n* = 12), or (3) pork sausage diet (Pork) (*n* = 12). The sausages were formulated from pork meat, pork fat and sunflower oil with added NaCl, phosphor, NaNO_2_, AIN76 vitamin mix (CA40077) and AIN76 mineral mix (CA79055) (Table S1). To the insect-substituted pork sausages 13.16 (*w/w* %) insect protein isolate from Alphitobius Diaperinus (~82% protein, Protifarm, Ermelo, Netherlands) was added. The energy and macronutrient content areprovided in Table 1.

The experimental diets and water were offered ad libitum and replenished twice a week during the whole study period. During the intervention period, food and water intake was monitored for each cage, and the bodyweight of each rat was measured at day 7, 14, 21, and 28.

### 2.2. Sample Collection

Fecal samples were collected from the individual rats both at baseline and at the end of the intervention. After the four weeks of intervention, the rats were anesthetized with fentanyl/fluanison (Hypnorm^®^, Skanderborg Pharmacy, Skanderborg, Denmark) and midazolam (5 mg/mL, Accord Health Care, Solna, Sweden) (each diluted 1:1 with sterile water prior to mixing; 0.2 mL/g body weight), heart blood was collected in heparin tubes and the rats were finally euthanized by heart blood bleeding under supplementary fentanyl/fluanison/midazolam. Plasma was obtained by centrifugation at 10,000× *g* for 5 min. Samples of stomach content, small intestinal content and colon content were carefully collected from the rats. All samples were snap-frozen in liquid nitrogen and stored at −80 °C until further analysis.

### 2.3. Sample Preparation for ^1^H-NMR Spectroscopy

Samples of stomach content, small intestinal content, colon content and feces were weighted, thawed and diluted 1:9 with ddH_2_O. The samples were whirl-mixed until they were dissolved, then centrifuged at 14,000× *g* for 10 min at 4 °C and the supernatant transferred to a new tube. Prior to this, 0.5 mL 10 K Amicon Ultra centrifugal filter units (Merck Millipore Ltd., Cork, Ireland) were prewashed four times with ddH_2_O. Then the supernatant was centrifuged at 14,000× *g* for 2 h at 4 °C before the supernatant was transferred to the filters. The samples were centrifuged at 14,000× *g* for 1 h at 4 °C. A volume of 400 µL of the resulting filtrate was transferred to a 5 mm NMR tube, together with 200 µL D_2_O and 50 µL phosphate buffer (8.66 % *w/v* K_2_HPO_4_, 1.812 % *w/v* NaH_2_PO_4_, H_2_O) containing sodium trimethylsilylpropanesulfonate (DSS) (0.23 % *w/v* DSS).

Plasma samples were thawed and 500 µL transferred to prewashed 10 K Amicon Ultra centrifugal filters followed by centrifugation at 14,000× *g* for 1 h at 4 °C. A volume of 500 µL filtrate was transferred to 5 mm NMR tubes with 25 µL D_2_O with 0.05 % Trimethylsilyl-propanoic acid (TSP), 75 µL D_2_O and 100 µL phosphate buffer (300 mM Na_2_HPO_4_).

### 2.4. H-NMR Spectroscopy

^1^H-NMR spectroscopy was conducted using a Bruker Avance III 600 MHz spectrometer operating at a ^1^H frequency of 600.13 MHz with a 5 mm ^1^H TXI probe (Bruker BioSpin, Rheinstetten, Germany). ^1^H-NMR spectra were obtained at 298 K using a one-dimensional (1D) nuclear overhauser enhancement spectroscopy (NOESY) preset pulse sequence (noesypr1d) to ensure water suppression. The acquisition parameters used were: 128 scans (NS), spectral width (SW) = 7289 Hz (12.15 ppm), acquisition time (AQ) = 2.25 s, 32 768 data points (TD), and relaxation delay (D1) = 5 s. Prior to Fourier transformation, the free induction decays (FIDs) were multiplied by a line-broadening function of 0.3 Hz. The spectra obtained were subjected to baseline correction and phase correction in TopSpin 3.0 (Bruker BioSpin, Billerica, MA, USA).

### 2.5. Metabolome Analysis

The ^1^H-NMR spectra were referenced to either TSP or DSS (0.00 ppm) and corrected for chemical shift by the interval correlation-shifting algorithm, icoshift [21] using MATLAB R2018b (Mathworks Inc., Natick, MA, USA). The spectral regions at the higher and lower chemical shift ranges without resonances and the spectral region containing the residual water signal were removed. Thereafter the spectral data were normalized to total area of the spectrum and binned into regions of 0.01 ppm. Multivariate data analysis was performed using SIMCA 16.0 (Sartorius Stedim Data Analytics AB, Umeå, Sweden), while Chenomx NMR Suite 8.13 (Chenomx Inc., Edmonton, Canada) was used for metabolite assignment and quantification using TSP or DSS as internal quantification standards. For plasma, the quantified concentrations were normalized to the sum of total quantified metabolites and are therefore relative values. Data were Pareto-scaled before principal component analysis (PCA) was conducted to examine variations in the data and possible differences between the diets. In addition, orthogonal projection to latent structures discriminant analysis (OPLS-DA) was performed to investigate the differences in the metabolome between the different diet groups. For each data group comparison, cross validation with seven cross validation groups was conducted and the Q_2_-value describing the predictive ability of the model was calculated. Finally, the metabolic differences discriminating the experimental diet groups were elucidated by S-line plot.

### 2.6. Gut Microbiota Analysis

Total DNA was extracted from the feces using Bead-Beat Micro AX Gravity Kit (A&A Biotechnology, Gdynia, Poland) according to the instructions of the manufacturer (bead beating 2 times 30 s, at 6.5 M/s in a FastPrep-24 (MP Biomedicals, Irvine, California, USA). The prokaryotic microbial community was characterized using 16S rRNA gene amplicon profiling of the V3-region (Illumina NextSeq-based), as described previously [22]. The zOTU table was constructed using the UNOISE3 pipeline [23], and SINTAX [24] was used to predict taxonomy. Downstream data analysis in microbial communities was performed using packages Phyloseq [25] and Vegan [26] in R (version 4.0.2). For α-diversity analysis, Shannon index was calculated. For β-diversity analysis, the Bray-Curtis method was used to generate the dissimilarity matrix, which was subsequently used in principal coordinate analysis (PCoA).

### 2.7. Ethical Statement

The rat intervention study was carried out in accordance with Directive 2010/63/EU of the European Parliament and of the Council of 22 September 2010 on the protection of animals used for scientific purposes, as well as the Danish Animal Experimentation Act (LBK 474 15/05/2014). Specific approval was granted by the Animal Experiments Inspectorate under the Ministry of Environment and Food in Denmark (License No 2017-15-0201-01262).

### 2.8. Statistical Analysis

To identify significant differences in the metabolites among the diet groups, one-way analysis of variance (ANOVA) was applied. To correct for multiple testing, false discovery rate (FDR) by Benjamin and Hochberg was applied, and q-values < 0.05 were considered significant. If a significant difference was found, the experimental groups were compared by Tukey’s honestly significant difference test, here a *p*-value < 0.05 were considered significant. Wilcoxon test was used to compare α-diversity differences. The component differences of different groups in PCoA were calculated by PERMANOVA tests. ANCOM [27] was used to detect differentially abundant taxa. The correlations between metabolites and differentially abundant bacteria genera were calculated by Pearson correlation, only metabolites and bacteria genera with at least one correlation coefficient larger than 0.7 were kept for downstream analysis.

## 3. Results

### 3.1. Feed Intake and Growth Is Not Changed by Insect Replacement

**Figure 1:**
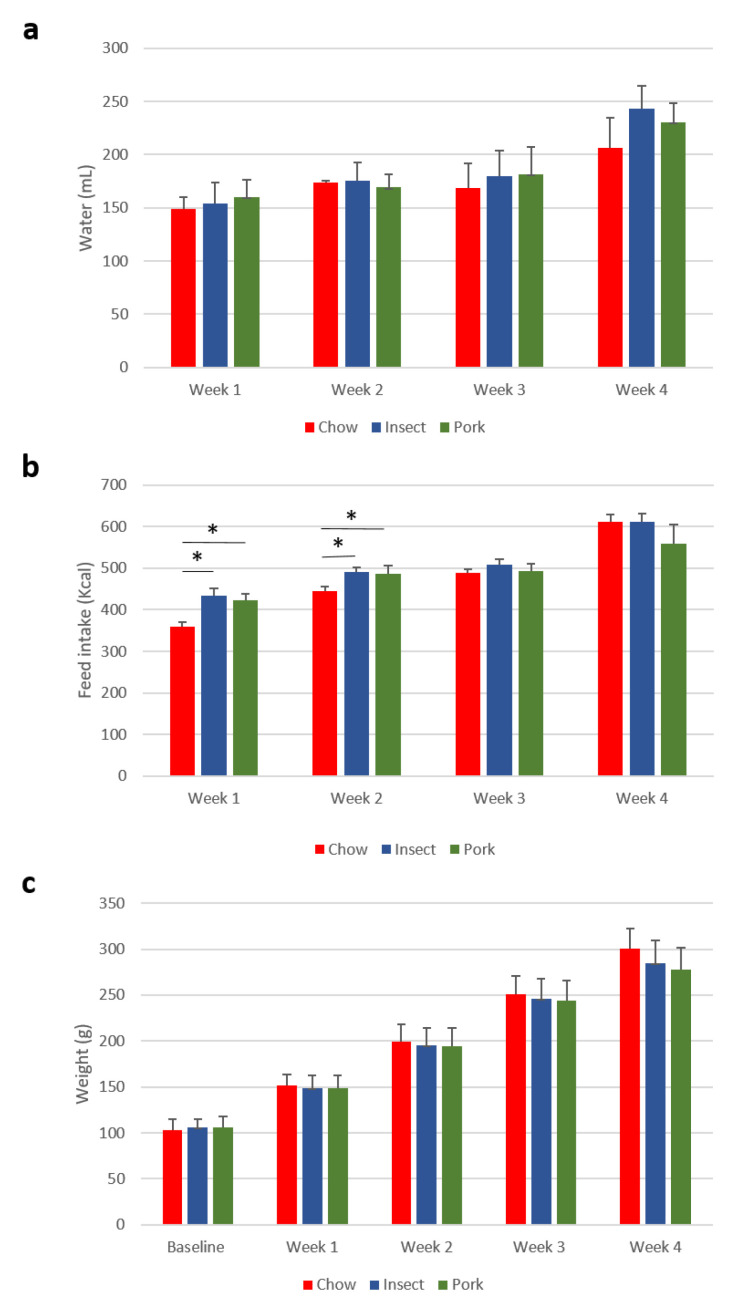
Water consumption, feed intake and body weight of the rats during the intervention period. (**a**) Water consumption (**b**) feed intake per week, and (**c**) the body weight of the rats was measured each week doing the intervention period. Data were analyzed by one-way ANOVA, values within the same cluster not sharing a common letter are significant different (*p* < 0.05). Chow diet (Chow, *n* = 6, red), insect-substituted pork sausage diet (Insect, *n* = 12, blue or pork sausage diet (Pork, *n* = 12, green). The groups found to be significantly different by ANOVA (*p*-value > 0.05) are marked with a star (*).

In the present study rats were fed ad libitum exclusively with one of the three diets: chow diet (Chow), insect-substituted pork sausage diet (Insect) or pork sausage diet (Pork) for a period of 4 weeks. Water consumption and feed intake were registered each week (Figure 1). No significant difference in water consumption was observed between the groups. In Weeks 1 and 2, the feed intake was significantly higher in the two sausage groups compared to the chow diet, but this was not reflected in the body weight of the rats where no significant differences among diet groups were observed. Similarly, no significant difference was found in the body weight after the four weeks of intervention.

### 3.2. Gut Microbiome (GM) Composition and Diversity

For alpha diversity analysis, the Shannon diversity values were calculated both before and after diet intervention and between the different diets after intervention. The diet intervention resulted in a significantly lower Shannon index after the pork diet (*p* = 0.023), and chow diet (*p* = 0.0087), while no significant difference was observed after the insect diet intervention (Figure S1A). The Shannon diversity showed no significant difference between the three diet groups after the intervention (Figure S1B).

**Figure 2:**
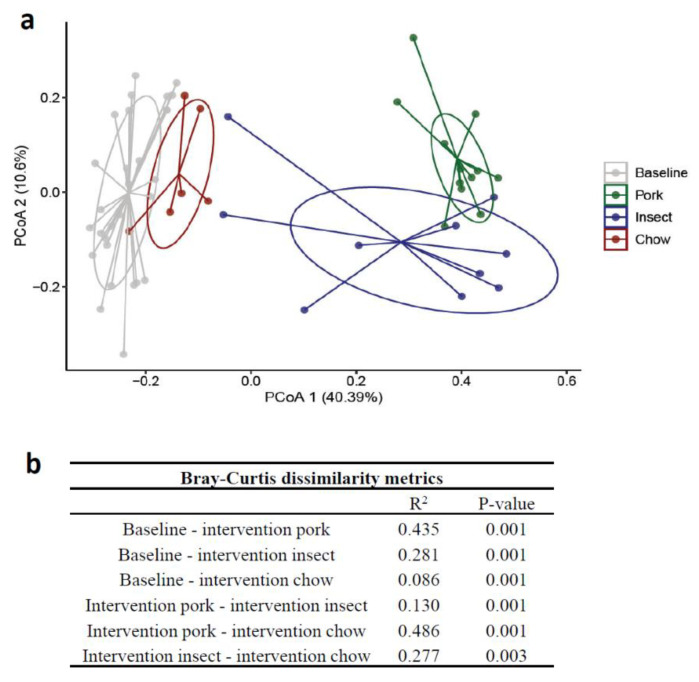
Beta diversity of the gut microbiota composition at baseline and after the three intervention diets. The gut microbiota composition was analyzed by 16S rRNA sequencing. PCoA plot of Bray-Curtis dissimilarity metrics of the gut microbiota composition comparing baseline (gray), pork sausage diet (*n* = 12, green), insect-substituted pork diet (*n* = 12, blue) or chow diet (*n* = 6, red) group (**a**). Statistical values of Bray-Curtis dissimilarity metrics for all comparisons (**b**).

Bray-Curtis dissimilarity metrics (Figure 2A) showed that all interventions were significantly different form baseline (*p* = 0.001). In addition, the three different diets resulted in significantly different GM communities with the chow intervention resembling the baseline samples more as compared to the two sausage intervention groups (Figure 2B).

**Figure 3:**
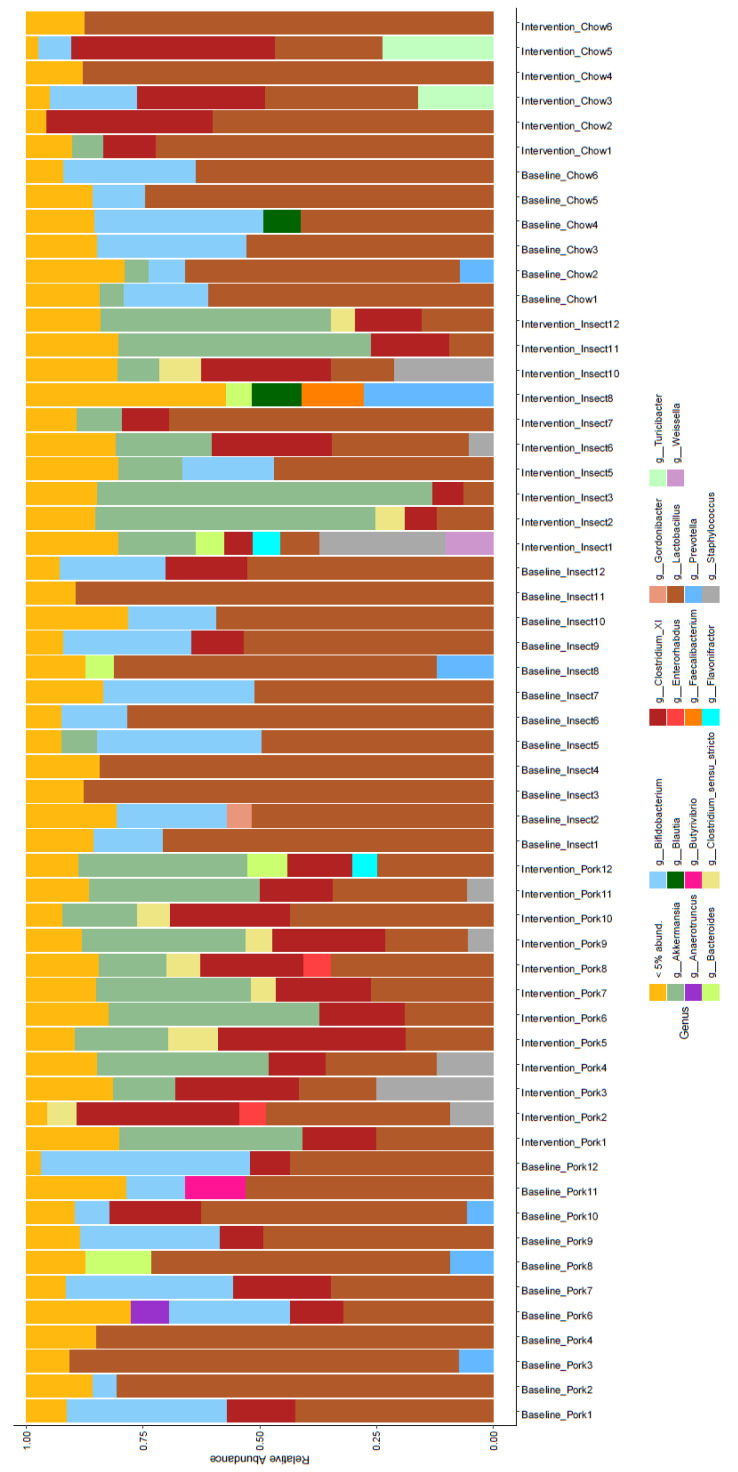
Gut microbiota composition at genus level for the individual rats before and after intervention diet. The gut microbiota composition were analyzed by 16S rRNA sequencing. Gut microbiota composition are shown for the individual rat at baseline and after intervention with chow diet (Chow), pork sausage diet (Pork) or insect-substituted pork diet shown as the relative abundance at genus level.

The GM of the individual rats before and after intervention was examined (Figure 3). Baseline samples were characterized by high relative abundance of *Lactobacillus* and *Bifidobacterium*. Additionally, 12 of the 29 rats were also colonized by *Clostridium cluster XI* at baseline. For the chow diet group, the microbial composition changed only marginally during the intervention. The GM remained dominated by *Lactobacillus*, while *Clostridium cluster XI*, *Bifidobacterium* and *Flavonifacter* also were present in some animals. The GM of rats in the pork diet intervention group was dominated by *Lactobacillus*, *Clostridium cluster XI* and *Akkermansia*. The GM of rats in the insect diet intervention group were similar to the pork diet group dominated by *Lactobacillus*, *Clostridium cluster XI* and *Akkermansia*. One rat (Number 8) showed a different GM pattern than all other rats. Changes of abundance at genus level after intervention diet are shown in detail in Figure S2.

### 3.3. Dietary Modulations of Metabolomes

In general, PCA scores plot of the metabolomics data obtained from the various sample types collected through the gastrointestinal tract (stomach content, small intestine content, colon content) revealed a clear grouping of the three intervention diets (Figure S3). OPLS-DA models were constructed for the pairwise comparison of pork sausage diet and insect-substituted pork diets to further investigate the metabolites responsible for the diet differentiation.

**Figure 4:**
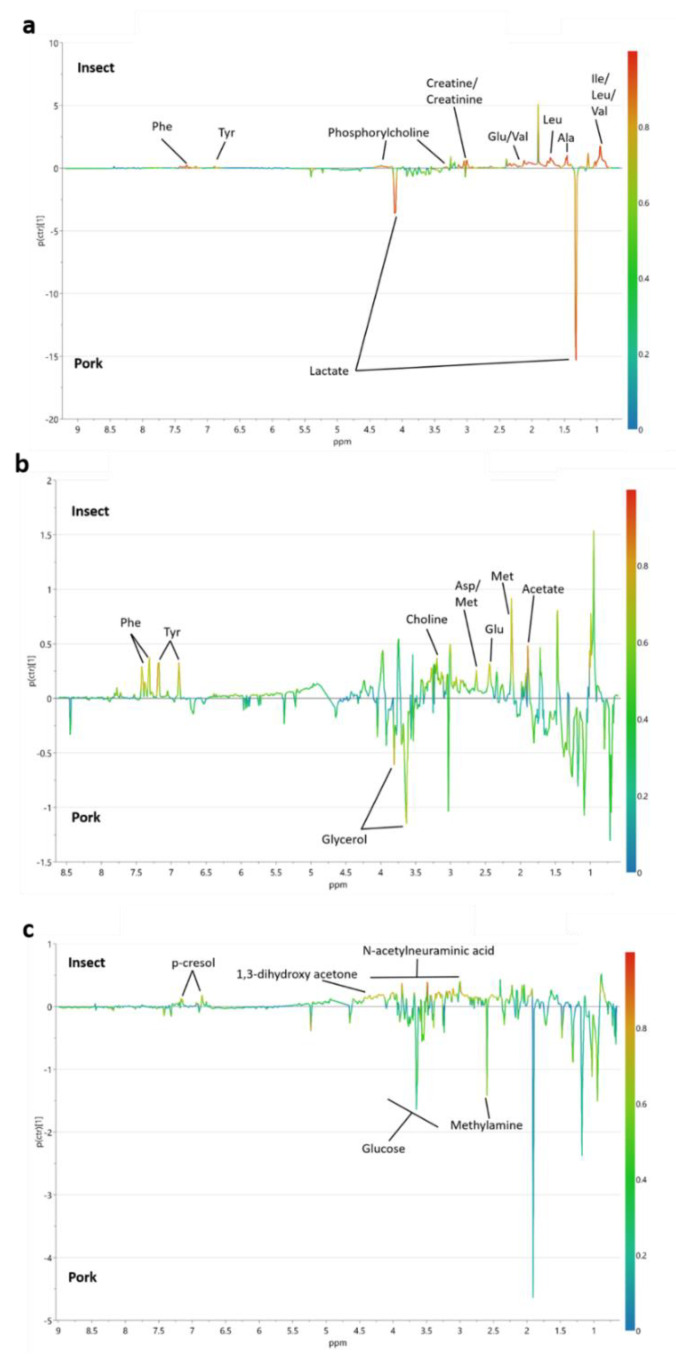
S-line plots from orthogonal partial least squares discriminant analysis of ^1^H NMR spectroscopic data on insect-substituted pork sausage diet (Insect) versus pork sausage diet (Pork) analysis. Stomach content, Q^2^ = 0.96 (**a**), small intestinal content, Q^2^ = 0.67 (**b**) and colon content, Q^2^ = 0.59 (**c**).

Pork sausage diet was associated with a higher concentration of lactate in the stomach content, while the insect diet resulted in a higher concentration of several metabolites including different amino acids such as glutamate, alanine, the branched-chained amino acids isoleucine, leucine, and valine and the two aromatic amino acids tyrosine and phenylalanine (Figure 4A). Increased levels of creatine, creatinine, phosphorylcholine were also accompanied with the insect diet (Figure 4A). The corresponding OPLS-DA score plot is shown in Figure S4A.

For OPLS-DA on metabolomics data from the small intestinal content, the S-line plot revealed that insect diet was associated with a higher concentration of choline and acetate as well as the amino acids aspartate, methionine, glutamate and the aromatic amino acids tyrosine and phenylalanine in small intestinal content compared to pork diet (Figure 4B). The OPLS-DA score plot is shown in Figure S4B.

For colon content, a S-line plot from an OPLS-DA model examining the metabolites discriminating the insect-substituted pork diet from the pork diet indicated that the insect diet was related to a higher level of 4-hydroxyphenylacetate, 1,3-dihydroxyacetone and N-acetylneuraminic acid while pork diet was characterized by slightly higher glucose and methylamine levels (Figure 4C). The score plot from the OPLS-DA model is shown in Figure S4C.

A robust OPLS-DA model discriminating the metabolite profile of feces after pork or insect-substituted pork diet, respectively, could not be obtained (Q_2_ = 0.34, data not shown). 

**Figure 5:**
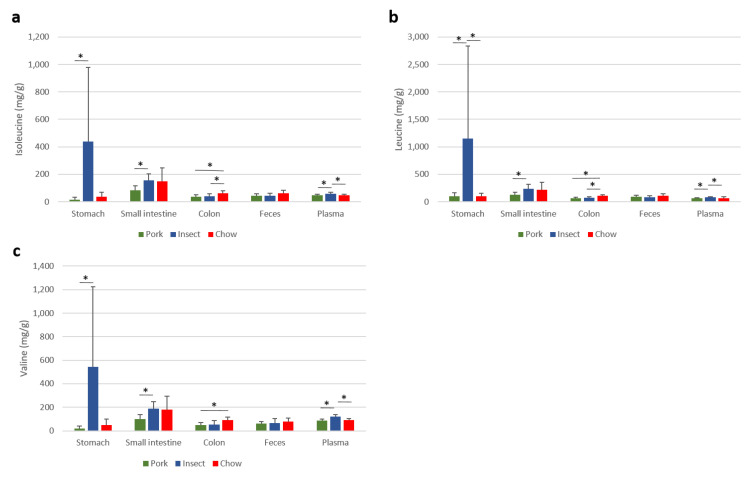
Concentration of branched chained amino acids (BCAA); Isoleucine (**a**), leucine (**b**) and valine (**c**) measured by ^1^H nuclear magnetic resonance spectroscopy. The concentrations found to significantly different by ANOVA (*p*-value > 0.05) are marked with a star (*). Values reported for gastrointestinal tract are in mg/g while plasma concentrations are expressed in relative values. Chow diet (Chow, *n* = 6, red), insect-substituted pork sausage diet (Insect, *n* = 12, blue) or pork sausage diet (Pork, *n* = 12, green).

The quantified amounts of the branched-chain amino acids (BCAAs) in plasma and through the gastrointestinal tract were examined (Figure 5). The relative concentrations of valine, leucine, and isoleucine in plasma were significantly higher for the insect diet compared to both the chow and the pork sausage diet groups. In addition, the amount of BCAA in stomach and small intestinal content was significantly higher after intake of the insect-substituted pork sausages compared to the pork sausage diet (Figure 5). In the colon content, the same trend was observed, however, differences between the insect and pork diets were not significant.

### 3.4. Correlation between GM and Metabolites

**Figure 6:**
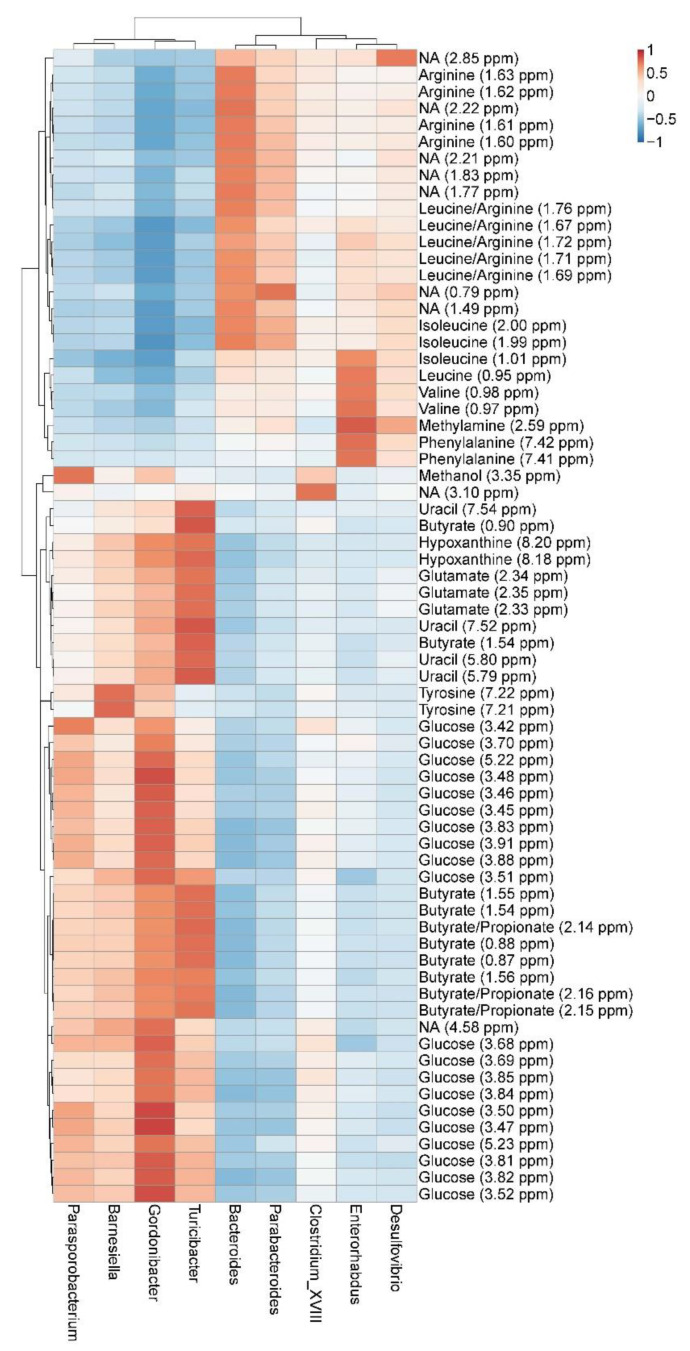
Heatmap of Pearson correlations between colon metabolite concentration and relative abundance of bacterial genera. The gut microbiota composition was analyzed by 16S rRNA sequencing while the metabolic pattern was analyzed by ^1^H NMR spectroscopy. Only metabolites and bacteria genera with at a correlation coefficient >0.7 are shown. The color intensity from blue to red corresponds to the correlation coefficient from −1 to 1.

Correlations between the GM and metabolites were investigated using Pearson correlation which revealed a separation of bacterial genera into two groups, with the relative abundance of each group being positively correlated to a range of metabolites, as shown by Figure 6. High relative abundance of *Parasporobacterium*, *Barnesiella*, *Gordonibacteria* and *Turicibacter* were positively correlated with glucose, butyrate, uracil, hypoxanthine, and glutamine. Methanol was correlated to the abundance of *Gordonibacter*, *Clostridium cluster XVIII* and especially *Parasporobacterium*, tyrosine was correlated to *Barnesiella* and *Gordonibacteria*, while high relative abundance of *Bacteriodes* and *Parabacteriodes* correlated to high concentrations of arginine and isoleucine. Finally, *Enterorhabdus* and *Desulfovibrio* relative abundance correlated to methylamine and the amino acids leucine, isoleucine, valine, and phenylalanine.

## 4. Discussion

In the present study, the impact of a partial substitution of meat protein with insect protein on the GM composition and metabolic processes in the gastrointestinal tract of healthy rats was investigated. Interventions with a traditional pork sausage and an insect-substituted sausage were compared with a conventional chow diet. The four week duration of the intervention was chosen to show the effects of prolonged ingestion of insect protein, mimicking an introduction of insect protein in a carnivore diet. If the changes shown in this paper will persist or increase with prolonged diet remains to be explored. Over four weeks, same energy intake and body weight gain was found irrespective of whether the rats were fed exclusively with pork protein or partly insect protein. 

Analysis of GM composition revealed pronounced changes in the gut microbiome composition following interventions with pork sausage and insect-substituted sausage when compared with rats remaining on a chow diet (Figure 2 and Figure 3). This may be expected considering that interventions with the carnivore diets (sausage products) were associated with changes in macronutrient composition. Interestingly, when comparing the GM after the interventions, Bray-Curtis dissimilarity metrics also revealed differences in GM composition between the pork diet and insect-substituted diet, respectively. Consequently, inclusion of insect as a protein-source in a carnivore diet impacted the GM to an extent where, after four weeks of intervention, it was clearly different from the rats fed the meat-based diet. A human study investigated intake of 25 g cricket powder per day for a two-week period where the cricket powder replaced cocoa powder and purple corn meal in a breakfast meal [18]. This replacement resulted in an increased fat and protein content and a lower carbohydrate content in the insect meal, but only resulted in smaller differences in the GM composition between individuals assigned to one of the two diets. Stull et al. found that insect ingestion was associated with an increase in abundance of *Bifidobacterium animalis*. In the current study, no increase of *Bifidobacterium* upon insect inclusion was observed (Figure S2). This might be related to differences in the background diet and food components that insect replaces or in the ability of *Bifidobacterium* to colonize in rats and humans [28]. 

Concomitantly with analysis of the GM composition, metabolic activity in the gastrointestinal tract was assessed through the application of nuclear magnetic resonance (NMR)-based metabolomics, and uniting the metabolomes from blood, stomach content, small intestinal content, colon content end feces pointed at a distinct metabolization of insect protein compared with meat protein in the gastrointestinal tract. The two carnivore diets: pork and insect, had metabolite profiles markedly distinct from the standard chow diet, which probably is a result of the differences in the macronutrient composition of the diets. The chow diet had a considerably higher content of carbohydrates and a lower amount of fat compared to the two carnivore diets. This also corresponds to earlier findings that high-carbohydrate-low-fat and low-carbohydrate-high-fat diets can be discriminated based on the metabolic profile of the plasma [29]. 

In the small intestine, ingestion of insect protein was associated with a larger amount of the aromatic amino acids tyrosine and phenylalanine compared with ingestion of meat protein. These aromatic amino acids are known to exert a high capability for stimulating fermentation in the gut [30]. In colon content, 4-hydroxyphenylactate was increased after ingestion of insect diet compared to pork diet. Further, 4-hydrophenylactate is involved in tyrosine metabolism and can most likely be linked to the higher concentration of tyrosine in the small intestine. Correlation analysis between GM and metabolites revealed that tyrosine levels in colon content correlated with the relative abundance of *Barnesiella* and *Gordonibacteria*. Thus, differences in tyrosine metabolism in the gut upon insect ingestion can possibly be ascribed to changes favoring the abundance of these specific bacteria. 

In blood plasma, higher levels of all branched-chain amino acids (BCAAs) leucine, isoleucine and valine were found in rats ingesting the insect-substituted feed compared with rats ingesting the conventional meat product (Figure 5). These results corroborate a former study in pigs, which found lower ileal digestibilities of amino acids for diets including insect meal compared with a control diet [16]. Even though it is not possible to confirm that these BCAAs in plasma are of microbial origin, this appears plausible as the two carnivore diets had a similar content of BCAAs. Other studies have also suggested that it is the overall dietary patterns rather than the dietary intake of BCAAs that contributes to BCAA plasma concentrations [31,32]. Levels of circulating BCAAs have been linked to an increased future risk of type 2 diabetes [33]. Consequently, the present findings might have implications for human health, but further studies are warranted to establish a potential association between insect intake and type 2 diabetes risk.

Recently, there is an intense focus on identifying new dietary protein sources as part of the great food transformation introduced in an EAT Lancet report [34]. The intention is that new foods sources should nurture human health and support environmental sustainability. The present study reveals the necessity of establishing actions to thoroughly examine how new dietary sources, commenced for sustainability reasons, impact endogenous metabolism and GM to capture their true value in terms of nurturing human health. Collectively, our data suggest that insect protein ingestion results in a different pattern of protein residues and metabolism in the large intestine and apparently an increased host absorption of generated BCAAs. Thus, while insect protein instantly appears as an attractive replacer of meat from a sustainable point of view, our study shows that it is important to approach new protein sources with a holistic approach to capture effects with implications for both human health and sustainability.

## 5. Conclusions

The present study shows that partial substitution of meat with insect protein in a traditional pork sausage over four-weeks, with same energy intake and body weight gain influence the gut microbiota composition as well as the plasma and gastrointestinal metabolite profile. Our data indicate that protein residues comes into the colon after inclusion of insect protein in diet compared to a diet exclusively containing pork meat protein. The increased amount of protein residues in the colon was reflected in a different metabolization of aromatic amino acid residues. Insect ingestion was also accompanied by a host absorption of generated BCAAs as reflected in higher plasma concentrations of these amino acids. Our study thus proposes that introduction of insects in a common Western omnivore diet alters the gut microbiome with consequences for endogenous metabolism.

## Figures and Tables

**Table 1:** Nutrient content of experimental diets.

	Pork Sausage Diet	Insect-Substituted Pork Sausage Diet	Chow Diet
Energy [Kcal/100 g]	288	286	319
Fat [g/100 g]	24.4	24.5	4.1
Carbohydrates [g/100 g]	4.2	1.5	40.8
Protein [g/100 g]	12.9	14.8	19.2

## Data Availability

Authors will make data available upon request.

## Supplementary Materials

The following are available online at https://www.mdpi.com/article/10.3390/foods10081814/s1, Table S1: Formulation of sausages diets. Ingredients added to common sausage emulsion of pork meat, pork fat and water, Figure S1: Alfa diversity of the gut microbiota composition at baseline and after the tree intervention diets. The Shannon index at baseline and after diet intervention (4 weeks) with either pork sausage diet, insect-substituted pork diet or chow diet (A). Statistical values of Shannon index for all comparisons (B), Figure S2: Change in abundance of different bacterial genera in the gut microbiota after diet intervention. The abundance at genus level are shown before (baseline) and after the different diet interventions (Intervention) with (A) pork sausage (pork), (B) insect-supplemented pork sausage (insect) or (C) chow diet (chow), Figure S3: Score plot of principal component analysis of stomach content. The first two principal components of rats on chow diet , pork sausage diet (blue) or insect-substituted pork sausage diet (green). The first principal component explaining 51% of the total variance separates the groups and by also taking the second component, explaining 27.5% of the variance, into account a clear separation is observed between the three groups, Figure S4: Score plot of OPLS-DA of stomach content of rats on insect-substituted pork sausage diet (green) versus pork sausage diet (blue). Q2 = 0.96, Figure S5: Score plot of principal component analysis of small intestinal content. The first two of three principal components of rats on chow diet , pork sausage diet (green) or insect-substituted pork sausage diet (blue). The first principal component (55.2%) separates the chow group from the others while the pork and insect diet groups are separated along both PC1 and PC2 (18.6%), Figure S6: Score plot of orthogonal partial least squares discriminant analysis of small intestinal content of rats on pork diet (green) versus insect-substituted pork sausage diet (blue). Q2 = 0.67, Figure S7: Score plot of principal component analysis of colon content. Score plot of principal component analysis with 7 principal components displaying the two first components of colon content of rats on chow diet , pork sausage diet (green) or insect-substituted pork sausage diet (blue). The first principal component (39.4%) separates the chow samples from the other groups while PC2 (14.8%) separates the pork from the insect group, Figure S8: Score plot of OPLS-DA of colon content of rats on pork diet (green) versus insect-substituted pork sausage diet (blue). Q2 = 0.59.
