# Supplementary material for: Partial Substitution of Meat with Insect (Alphitobius diaperinus) in a Carnivore Diet Changes the Gut Microbiome and Metabolome of Healthy Rats
Source: Foods. 2021 Aug 5;10(8):1814. doi: 10.3390/foods10081814 (PMC8393340; doi:10.3390/foods10081814)
Supplement: Supplementary file 1 [file foods-10-01814-s001.zip › foods-1311373-SI.pdf]

Supplementary material

Table S1: Formulation of sausages diets. *Ingredients added to common sausage emulsion of pork meat, pork fat and water*

	**Pork sausage**	**Insect-substituted pork sausage**
**Sunflower oil [mL/kg]**	62.4	65.8
**NaCl [g/kg]**	11.2	11.8
**Phosphor [g/kg]**	4.7	4.9
**$NaNO_2$ [g/kg]**	7.5	7.9
**AIN76 mineral mix (TD79055) [g/kg]**	18.7	19.7
**AIN76 vitamin mix (CA40077) [g/kg]**	5.9	6.2
**Insect protein isolate [g/kg]**	-	131.6

A

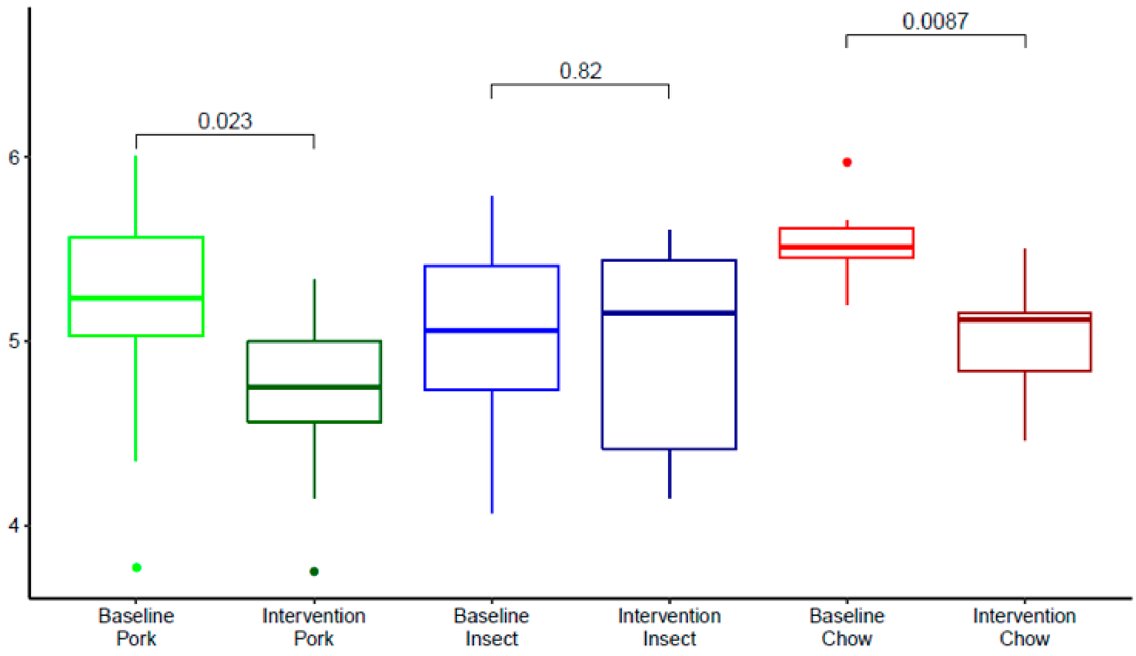

B

Shannon index	
	H'
Baseline pork - intervention pork	0.023
Baseline insect - intervention insect	0.82
Baseline chow - intervention chow	0.0087
Intervention pork - intervention insect	0.25
Intervention pork - intervention chow	0.12
Intervention insect intervention chow	0.96

Figure S1: Alfa diversity of the gut microbiota composition at baseline and after the tree intervention diets. *The Shannon index at baseline and after diet intervention (4 weeks) with either pork sausage diet, insect-substituted pork diet or chow diet (A). Statistical values of Shannon index for all comparisons (B).*

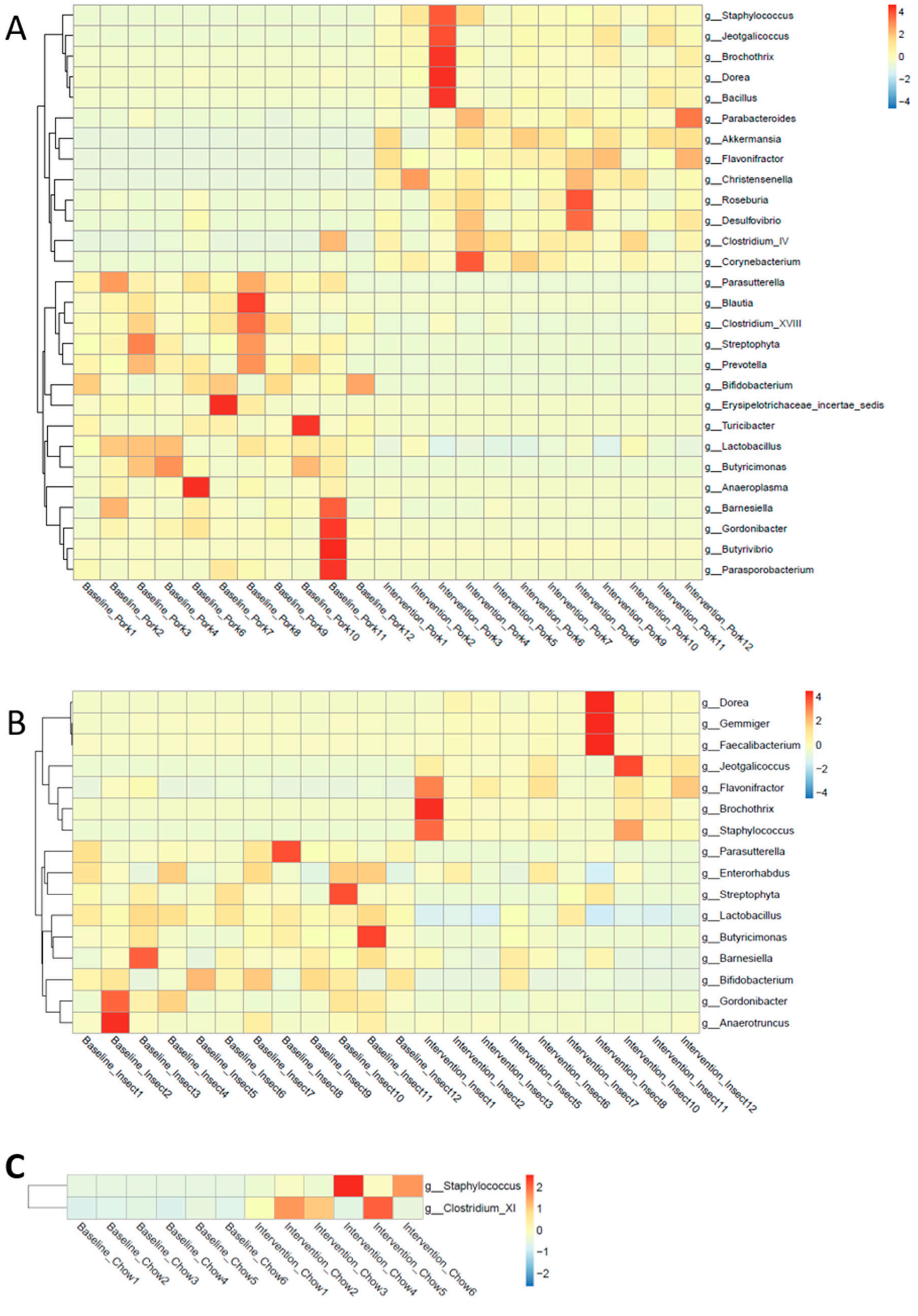

Figure S2: Change in abundance of different bacterial genera in the gut microbiota after diet intervention. *The abundance at genus level are shown before (baseline) and after the different diet interventions (Intervention) with (A) pork sausage (pork), (B) insect-supplemented pork sausage (insect) or (C) chow diet (chow).*

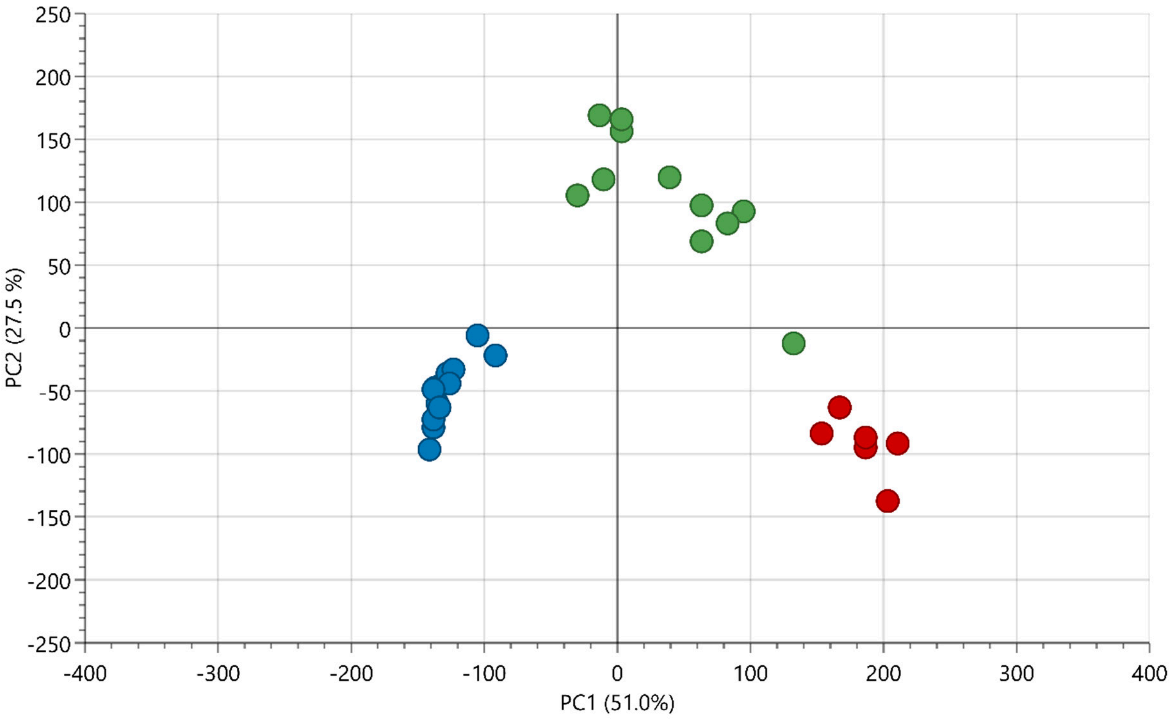

Figure S3: Score plot of principal component analysis of stomach content. *The first two principal components of rats on chow diet (red), pork sausage diet (blue) or insect-substituted pork sausage diet (green). The first principal component(PC) explaining 51 % of the total variance separates the groups and by also taking the second component, explaining 27.5 % of the variance, into account a clear separation is observed between the three groups.*

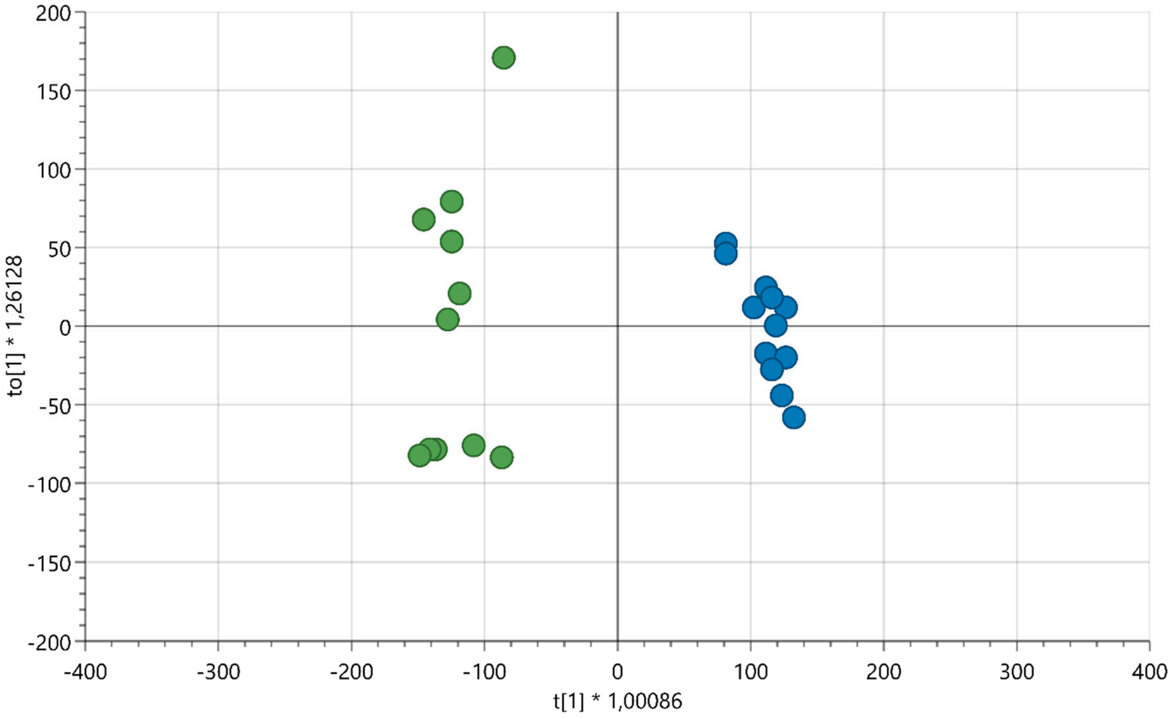

Figure S4: Score plot of *OPLS-DA* ***of stomach content of rats on insect-substituted pork sausage diet (green) versus pork sausage diet (blue).*** *Q^2=0.96.*

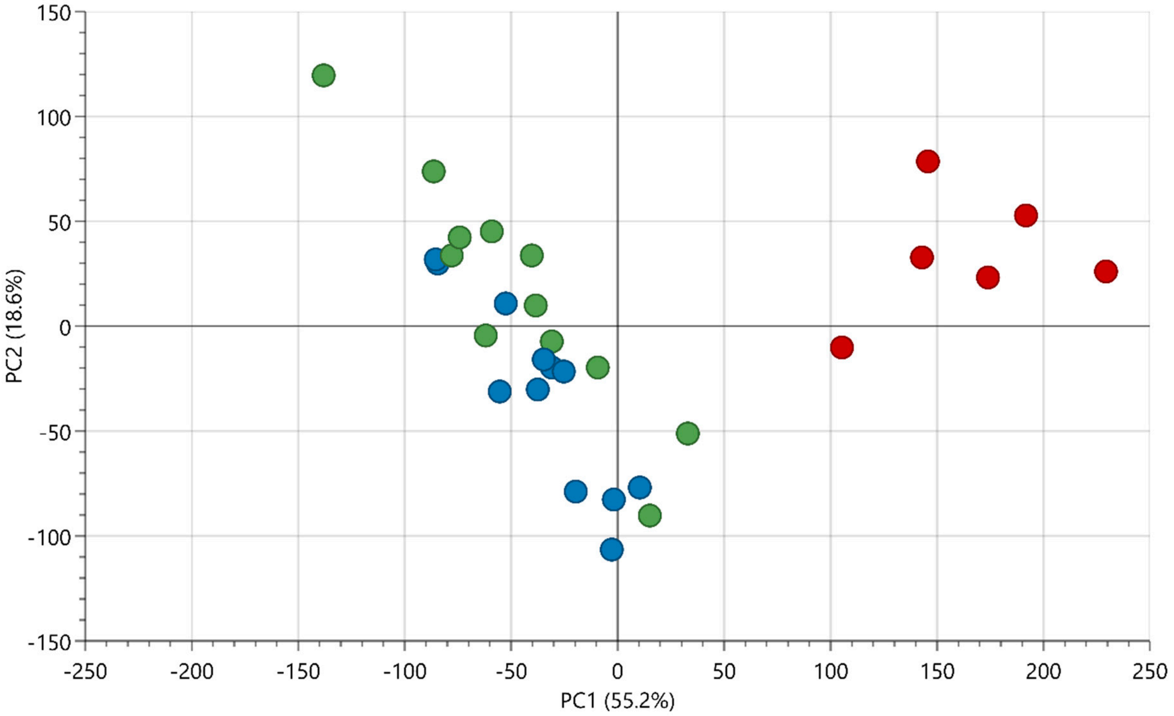

Figure S5: Score plot of principal component analysis of small intestinal content. *The first two of three principal components of rats on chow diet (red), pork sausage diet (green) or insect-substituted pork sausage diet (blue). The first principal component (PC) (55.2 %) separates the chow group from the others while the pork and insect diet groups are separated along both PC1 and PC2 (18.6 %).*

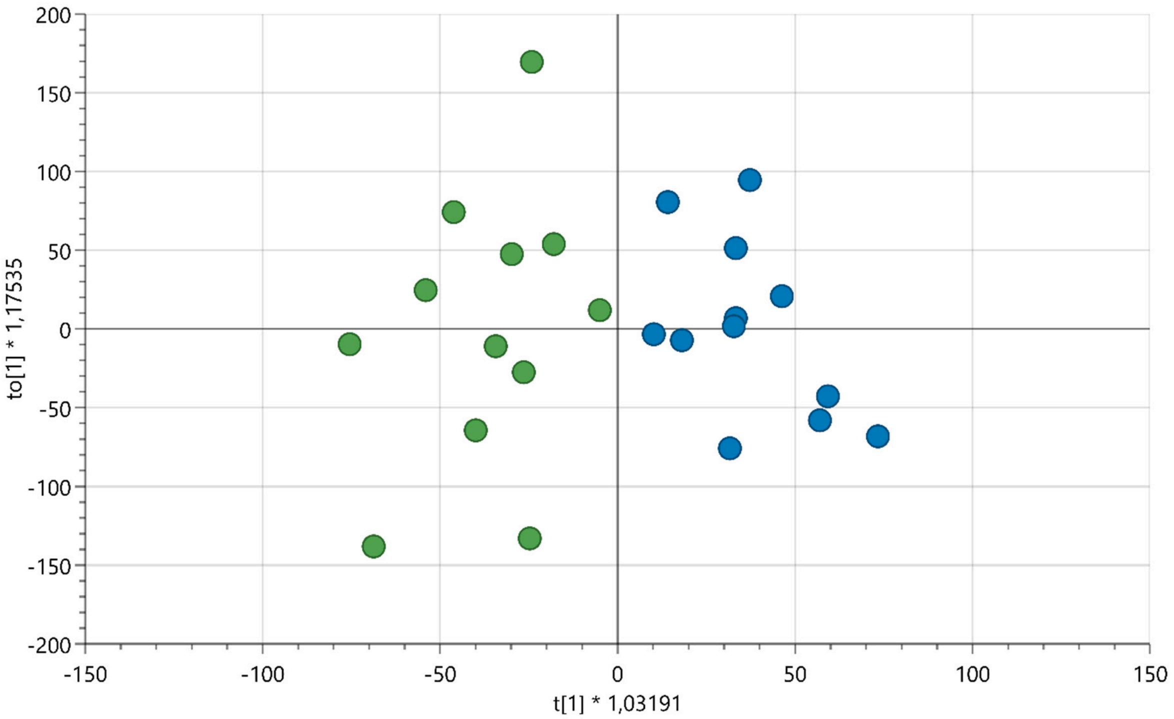

Figure S6: Score plot of orthogonal partial least squares discriminant analysis of small intestinal content of rats on pork diet (green) versus insect-substituted pork sausage diet (blue). *Q^2=0.67.*

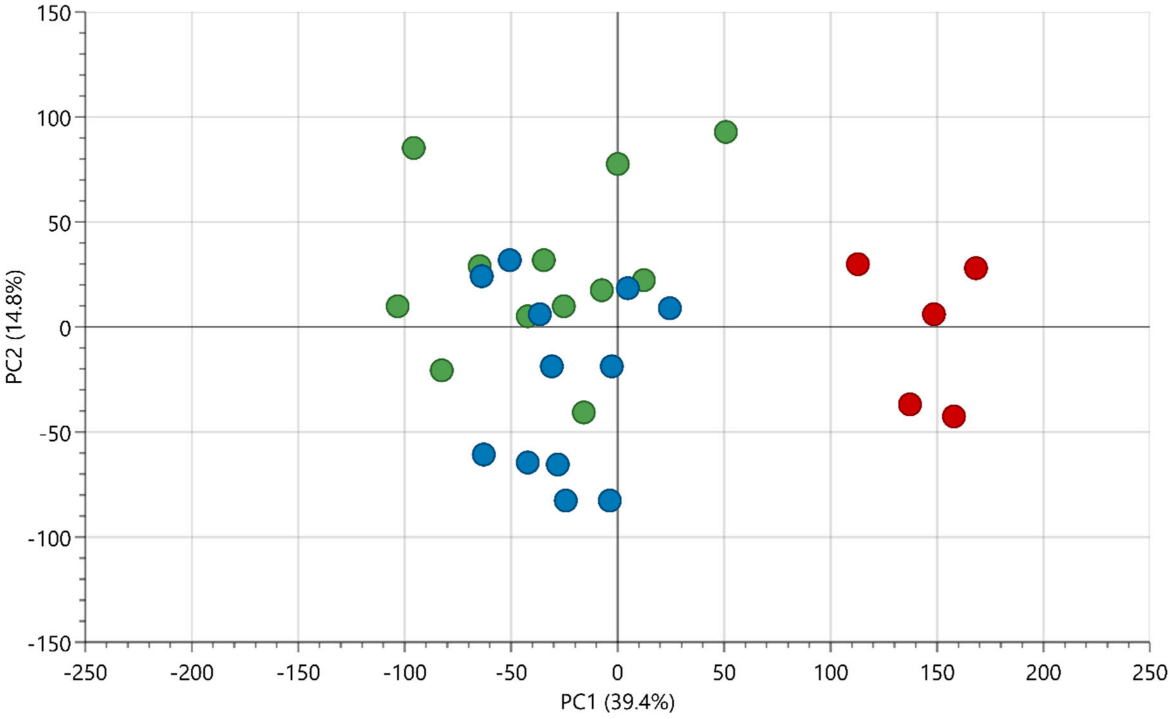

Figure S7: Score plot of principal component analysis of colon content. *Score plot of principal component analysis with 7 principal components displaying the two first components of colon content of rats on chow diet (red), pork sausage diet (green) or insect-substituted pork sausage diet (blue). The first principal component (PC) (39.4 %) separates the chow samples from the other groups while PC2 (14.8 %) separates the pork from the insect group.*

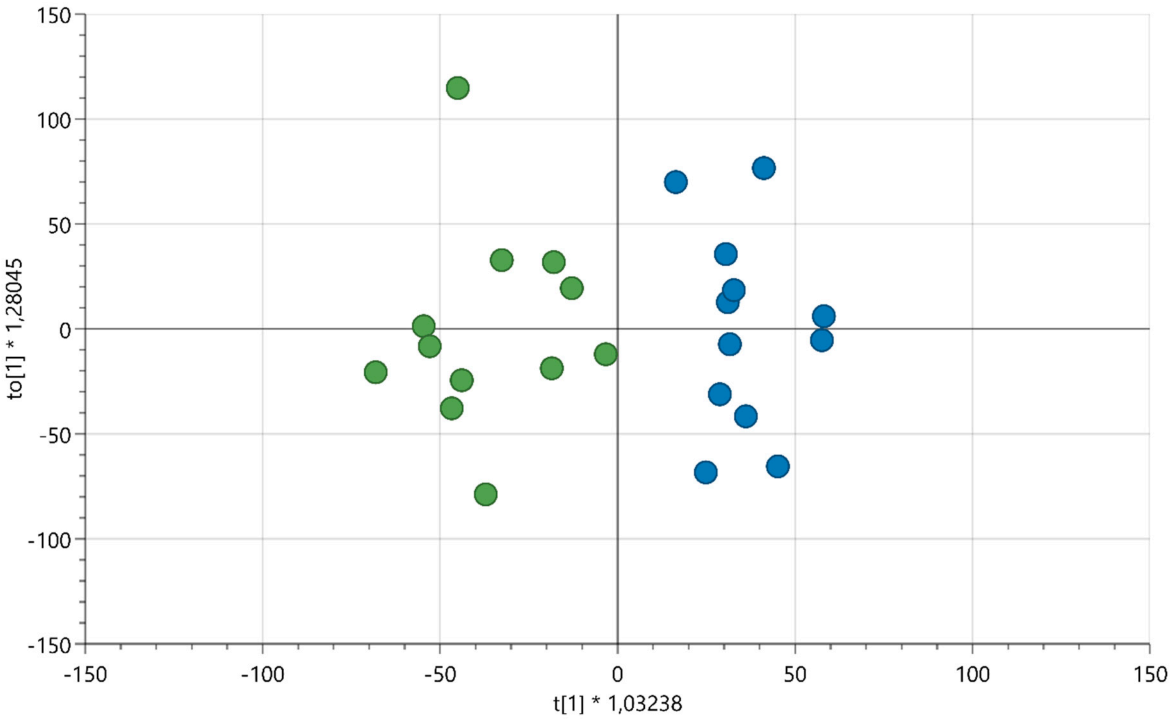

***Figure S8: Score plot of** OPLS-DA **of colon content of rats on pork diet (green) versus insect-substituted pork sausage diet (blue).** Q^2=0.59.*
